# Small-Bowel Neoplasms: Role of MRI Enteroclysis

**DOI:** 10.1155/2016/9686815

**Published:** 2015-12-24

**Authors:** Angela Faggian, Maria Rosaria Fracella, Grazia D'Alesio, Maria Eleonora Alabiso, Daniela Berritto, Beatrice Feragalli, Vittorio Miele, Francesca Iasiello, Roberto Grassi

**Affiliations:** ^1^Institute of Radiology, Second University of Naples, Piazza Miraglia 2, 80138 Naples, Italy; ^2^Department of Radiology, San Paolo Hospital, Contrada Caposcardicchio, 70123 Bari, Italy; ^3^Department of Radiology, IGEA Sant'Antimo, Italy; ^4^Department of Oral Science, Nano and Biotechnology, University G. d'Annunzio of Chieti-Pescara, Italy; ^5^Department of Emergency Radiology, S. Camillo Hospital, Circonvallazione Gianicolense 87, 00152 Rome, Italy

## Abstract

Small-bowel neoplasms are the 3%–6% of all gastrointestinal tract neoplasms. Due to the rarity of these lesions, the low index of clinical suspicion, and the inadequate radiologic examinations or incorrect interpretation of radiologic findings, a delay in diagnosis of 6–8 months from the first symptoms often occurs. Even if conventional enteroclysis and capsule endoscopy are the most common procedures used to accurately depict the bowel lumen and mucosal surface, their use in evaluating the mural and extramural extents of small-bowel tumors is limited. Instead multidetector computed tomographic enteroclysis and magnetic resonance enteroclysis have the potential to simultaneously depict intraluminal, mural, and extraintestinal abnormalities. In particular MR enteroclysis has an excellent soft tissue contrast resolution and multiplanar imaging capability. It can provide anatomic, functional, and real time information without the need of ionizing radiation. MR findings, appearances of the lesions, combined with the contrast-enhancement behavior and characteristic of the stenosis are important to differentiate small-bowel neoplasm from other nonneoplastic diseases.

## 1. Introduction

Small-bowel neoplasms are the 3%–6% of all gastrointestinal tract neoplasms, although the small-bowel represents 75% of the length and 90% of the mucosal surface of the gastrointestinal tract. They can develop from all the various tissue components of the wall: mucosa, submucosa, and muscle layers [1]. Patients may present obscure GI bleeding and nonspecific symptoms such as abdominal pain, nausea and vomiting, weight loss, diarrhea, anaemia, and intestinal obstruction. However, many patients may remain asymptomatic until the late stages of disease [2].

Due to the rarity of these lesions, the low index of clinical suspicion, and inadequate radiologic examinations or incorrect interpretation of radiologic findings, a delay in diagnosis of 6–8 months from the first symptoms often occurs, conditioning surgical therapy and survival of patients [3].

Conventional enteroclysis and capsule endoscopy are the most common procedures used to accurately depict the bowel lumen and mucosal surface, but their use in evaluating the mural and extramural extents of small-bowel tumors is limited. Multidetector computed tomographic (CT) enteroclysis and magnetic resonance (MR) enteroclysis have the potential to simultaneously depict intraluminal, mural, and extraintestinal abnormalities.

Multidetector CT enteroclysis involves the use of ionizing radiation, limiting repeated imaging, which is important in determining whether an area of intestinal narrowing is due to a contraction in the intestinal or to fixed strictures [2, 4–6]. MR enteroclysis has an excellent soft tissue contrast resolution, multiplanar imaging capability, and a lack of ionizing radiation. The possibility to repeat data acquisition over time and the ability to perform real time functional imaging permit functional evaluation of small-bowel mobility [7, 8]. The purposes of our study were to retrospectively evaluate the accuracy of MR enteroclysis, using histological findings as the reference standards, and to assess the interobserver variability for detection of small-bowel neoplasms.

## 2. Materials and Methods

### 2.1. Study Design and Population

Between March 2009 and December 2014 a retrospective study was performed evaluating exams of 67 patients (male/female ratio 3 : 1; mean age: about 57 years) with a clinical suspicion of intestinal neoplasia. Patients had already performed a gastroscopy and/or colonoscopy. Clinical suspicion was represented by intermittent bouts of intestinal obstruction and abdominal pain (N_9), obscure GI bleeding or chronic anemia (N_35), protein-losing enteropathy (N_7), and asthenia (N_16). All patients underwent MR enteroclysis. Diagnostic confirmation was obtained by histological examination of the surgical specimen or biopsy specimen or by follow-up with colonoscopy, videocapsule endoscopy, enteroclysis, or conventional enteroclysis RM after 6 months.

### 2.2. MR Enteroclysis

Three days before the exam each patient reduces or totally eliminates fiber, from the afternoon of the day before the exam, an isosmolar laxative (SELG ESSE 1000) diluted in 4 liters of water was prescribed, and only fluid diet was allowed.

MR imaging studies were performed with phased-array coil on a 1.5-T closed magnet (Magnetom Symphony, Siemens, Germany). In accordance with our institute guidelines, every patient received and signed written consent forms. After fluoroscopically guided nasojejunal intubation, while the patient lay prone inside the magnet, the small bowel is distended with 1,500–2,000 mL of polyethylene glycol- (PEG-) water solution using an electric infusion pump with a speed of injection of 120–150 mL/min. The MR protocol consists of MR fluoroscopy using RARE (T2-weighted half-Fourier rapid acquisition with relaxation enhancement) single-shot sequences in real time, starting at the beginning of the infusion and repeated every 8 seconds during normal breathing until the PEG-water solution reached the ascending colon and the entire small bowel was adequately distended. Then 20 mg of hyoscine butylbromide (Buscopan; Boehringer-Ingelheim, Ingelheim, Germany) was administered intravenously to reduce small-bowel peristalsis and prolong small-bowel distention and the MR examination was completed with cross-sectional imaging. Axial, sagittal, and coronal single-shot HASTE, TrueFISP with and without fat suppression (repetition time msec/echo time msec 3.6–3.8/1.5–1.7; matrix 192 × 340; section thickness/gap mm 5/0) were performed for morphological study of small bowel. Then VIBE T1 Flash 3D FAT-SAT sequences (repetition time msec/echo time msec, 3.24/1.24; field of view, 400 mm, even if it depends on the size of the patient; matrix, 288 × 512; flip angle, 10°; one signal is acquired; section thickness, 2.50 mm) in multiple breath-hold series repeated at least seven times in a row in expiratory apnea were obtained at 0°, 30′, 60′, 90′, and 120′ after contrast injection (0.1 mmol/kg gadolinium at 2 mL/sec). Diffusion-weighted MR imaging (DW-MRI) was also performed in the true axial plane using a single-shot spin-echo echo-planar imaging (SE-EPI) sequence with *b* values of 50, 400, and 800 s/mm^2^ (repetition time msec/echo  time msec, 5600/80; field of view, 500 mm, even if it depends on the size of the patient; matrix,   288 × 512; section thickness, 6 mm) in multiple breath-hold series repeated at least seven times in a row. Apparent diffusion coefficient (ADC) measurement by DW-MRI was done.

Images were analyzed by two experienced observer board-certified abdominal radiologists (Maria Rosaria Fracella and Roberto Grassi) with 25- and 30-year experience. Both reviewers were blinded to clinical details, results of previous investigations.

### 2.3. Statistical Analysis

For each reader, sensitivity, specificity, positive predictive value (PPV), negative predictive value (NPV), and the diagnostic accuracy have been calculated. Furthermore interobserver agreement was assessed (con il test *κ* di cohen) with *κ* statistics. A *κ* value greater than or equal to 0.75 was considered to represent excellent agreement.

## 3. Results

MR enteroclysis was successfully performed in all patients. For one reader MR enteroclysis revealed 24 lesions (35.8%) and 23 for the second one (34.3%). Diagnosis was confirmed in 23 patients. Malignant neoplasms were diagnosed in 17 cases: 3 adenocarcinomas, 6 lymphomas, 3 small-bowel metastases, 1 neuroendocrine tumor, and 4 GIST. Benign neoplasms were diagnosed in 6 cases: 2 leiomyomas, 1 adenoma, and 3 hamartomatous polyps.

False positives were due to two adhesions and a substenosis with wall thickening. False negatives were cases of hamartomatous polyps and jejunal metastases.

Sensitivity of MR enteroclysis in the diagnosis of small-bowel neoplasms in our sample data was 87.5% and 91.6%, while specificity was 93 and 97.6%, respectively, for readers 1 and 2 (Table 1).

There was excellent agreement between the readers, with a *κ* value > 0.9 for MR enteroclysis diagnosis of small-bowel neoplasm.

## 4. Discussion

The lack of ionizing radiation, the possibility of combining the morphologic information of cross-sectional imaging with functional information, the excellent soft-tissue contrast, and a relatively safe intravenous contrast agent profile make MR imaging the method of choice for the study of the small intestine. Moreover the opportunity of visualizing the entire thickness of the bowel wall and studying the surrounding structures makes MR imaging an excellent method not only for diagnosis but also for staging and prognosis [9–11]. Our results confirm that MR enteroclysis is an accurate modality with which to diagnose or exclude small-bowel neoplasms [1, 12]. Bowel cleansing and optimal distention of the small-bowel loops are crucial for the correct evaluation of the bowel wall because collapsed bowel loops can hide lesions or mimic disease by suggesting an abnormality-related thickened bowel wall in collapsed segments [13, 14].

**Figure 1:**
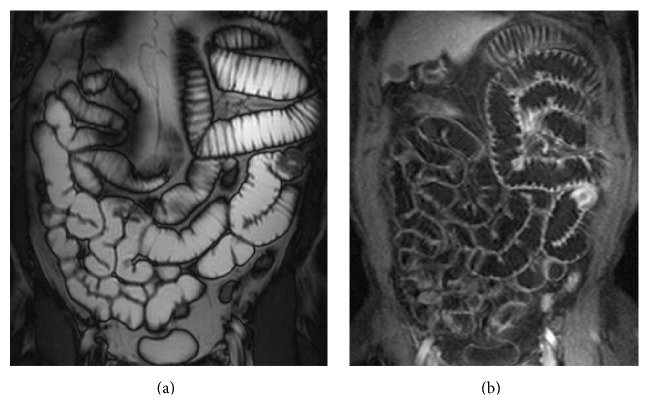
MRI, TrueFISP coronal sequence (a): a circumferential thickening protruding in the intestinal lumen with tendency to invagination is detected, in absence of local infiltration. In the VIBE sequences after the i.v. contrast medium administration (b), there is an inhomogeneous contrast enhancement. Definitive histology: adenocarcinoma.

**Figure 2:**
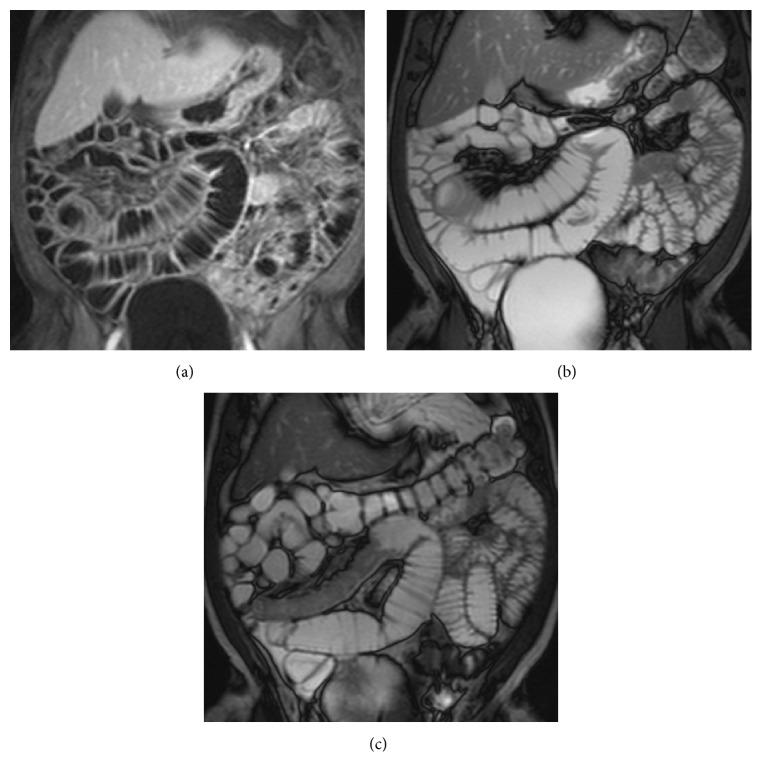
Lesion with nodular aspect protruding into the intestinal lumen with infiltrative growth. A significant desmoplastic reaction and fibrosis of adjacent loop are also present. Definitive histology: neuroendocrine tumor.

Small-bowel distention was obtained with nasojejunal. Although this procedure was not always well accepted by patients and was characterized by longer time of the examination, as well as the use of X-rays, it improves the quality of the investigation since distension of bowel loops is controlled and uniform in contrast to the result obtained with the administration of contrast per os during procedures of MR enterography (Figures 1 and 2) [1, 15]. The use of coronal single-shot spin-echo (MR fluoroscopy) sequences is important for determining the distensibility of narrowed areas and facilitating the differentiation of contractions from strictures in the evaluation of prestenotic dilatation, of small-bowel mobility. The excellent soft tissue contrast allows evaluation of the layers of the wall and then the mucosal, submucosal or extraparietal origin of the diseaseof the disease [1, 8, 16, 17]. Furthermore ADC measurement by DW-MRI provided useful information to better characterize small lesions. An additional result was the excellent interobserver agreement achieved with MR enteroclysis, which indicates that this procedure can enable reproducible evaluation of small-bowel abnormalities.

**Figure 3:**
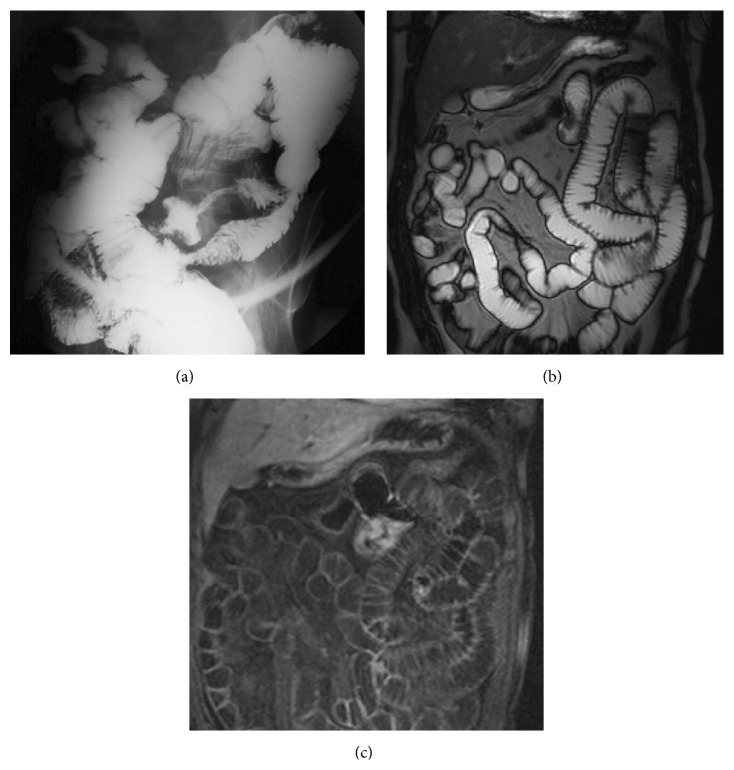
Lesion with infiltrative pattern, protruding into the intestinal lumen, with intense contrast enhancement in the VIBE sequences after the i.v. contrast medium administration (c). No evident lymphadenopathy. Definitive histology: lymphoma.

Lymphomas represented the most common malignant tumors in our results, even if in the literature it is reported that they are about 20% of primary malignancies of the small intestine (Figure 3) [10]. B-cell lymphoma (4 cases) and follicular lymphoma (2 cases) were identified in our sample data. The first were located in the distal ileum, in agreement with the most frequent site described in the literature [16, 18] and appeared as polypoid lesions that protruded into the lumen and in one case ulceration and fistula were associated. Follicular lymphoma was located in the duodenum and appeared as thick walls without proximal obstruction, as the neoplasm does not elicit a desmoplastic response, and discreet parietal enhancement after intravenous contrast medium.

Cases of primitive adenocarcinoma were all localized in jejunal (2 cases of proximal jejunum, 1 case of distal jejunum), even if the incidence is the highest in the duodenum [19].

Characteristics of these lesions were sub-stenosis and concentric wall thickening with length between 2.7 and 3.3 cm (2 cases) and irregular intraluminal vegetation (1 case), with moderate enhancement after administration of contrast medium. In all cases a marked restriction of the diffusion signal was observed. In one case there was perivisceral adenopathy. One neuroendocrine tumor was identified with the appearance of focal and asymmetric bowel-wall thickening in the medium ileum (maximum diameter of 3 cm) with desmoplastic reaction infiltrating the adjacent ileal loops.

Gist appeared as well-circumscribed masses with intramural submucosal location and exophytic growth in three cases; in one case mucosal association was present and in another case a focal intraluminal polypoid mass was identified. For all these lesions after intravenous administration of gadolinium the solid portions enhanced in a peripheral heterogeneous fashion.

**Figure 4:**
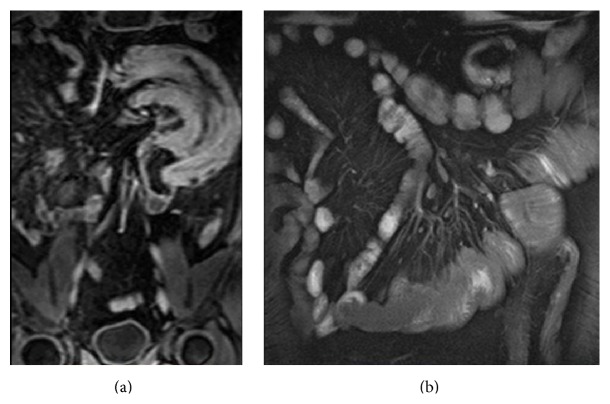
MRI, VIBE sequences after the i.v. contrast medium administration (a) and HASTE sequences (b): large mass with endophytic growth with intense and heterogeneous enhancement. Small lymph nodes are evident in the root of the mesentery. Definitive histology: metastases from melanoma.

Three metastases was observed in our sample data. Two of these were caused by haematogenous spread from small cells lung cancer and melanoma (Figure 4) and appeared as round polypoid mass; the last one was an intraperitoneal metastasis from colon cancer that appeared as thick walls on the antimesenteric border of the small-bowel wall. In these three patients lymph nodes were identified at the mesenteric root.

**Figure 5:**
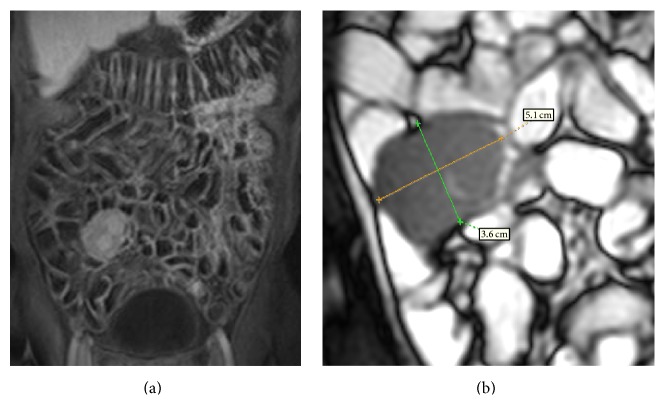
Round lesion, with regular contours, in patients with frequent occlusive syndromes. It shows moderate enhancement in postgadolinium sequences (a). Definitive histology: leiomyoma.

Leiomyoma (Figure 5) was identified as an oval mass with regular margins in the distal ileum and intense uniform enhancement. Adenoma and the hamartomatous polyps appeared as solid polypoid pedunculated masses, with regular margins and the maximum diameter of 2 cm.

## 5. Conclusion

According to the literature our results show that MR enteroclysis is an accurate modality for detecting small-bowel neoplasm. It can provide anatomic, functional, and real time information without the need of ionizing radiation. MR findings, appearances of the lesions, combined with the contrast-enhancement behavior and characteristic of the stenosis are important to differentiate small-bowel neoplasm from other nonneoplastic disease [1, 20, 21].

## Figures and Tables

**Table 1:** Results of MR enteroclysis in the diagnosis of small-bowel neoplasms in our sample data for readers 1 and 2.

	Reader 1	Reader 2
Number of true-positive cases	21	22
Number of false-positive cases	3	1
Number of true-negative cases	40	42
Number of false-negative cases	3	2
Sensitivity (%)	87.5	91.6
Specificity (%)	93	97.6
PPV (%)	87.5	95.6
NPV (%)	93	95.4
Diagnostic accuracy	91	95
interobserver agreement	*κ* value^*∗*^ > 0.9	

^*∗*^Interobserver agreement regarding lesion detection was excellent (*κ* > 0.85).

NPV: negative predictive value; PPV: positive predictive value.
